# Special Issue “Updates on Synthetic and Natural Antioxidants”

**DOI:** 10.3390/ijms27073102

**Published:** 2026-03-29

**Authors:** Izabela Sadowska-Bartosz, Grzegorz Bartosz

**Affiliations:** Laboratory of Analytical Biochemistry, Institute of Food Technology and Nutrition, Faculty of Technology and Life Sciences, University of Rzeszów, 35-601 Rzeszów, Poland; grzegorz.bartosz@gmail.com

## 1. Introduction

Antioxidants have been the subject of sustained research interest for years. The PubMed database returns over 836,000 results for the keyword “antioxidant,” while the number of results returned by Google Scholar approaches 3.9 million (23 March 2026). What is an antioxidant? The classical definition describes an antioxidant as “a substance that, when present at low concentrations compared with that of an oxidizable substrate, significantly delays or inhibits oxidation of that substrate” [1]. This definition, appropriate for simple chemical systems, is too narrow to encompass the broad range of effects observed at both cellular and organismal levels caused by compounds that are referred to as antioxidants in a broader, biological sense. The definition proposed by the Panel on Dietary Antioxidants and Related Compounds of the Food and Nutrition Board states that “a dietary antioxidant is a substance in food that significantly decreases the adverse effects of reactive oxygen species (ROS), reactive nitrogen species, or both on normal physiological function in human” [2]. One may still wonder if ROS and reactive nitrogen species complete the list of possible oxidants occurring in vivo. In our opinion, antioxidants can be defined as compounds that decrease (directly or indirectly) the extent of undesired oxidation reactions in a given system. This definition is generally applicable to living cells and organisms as well as food products and inanimate systems. It encompasses compounds that do not show direct antioxidant activity in vitro. As inflammation involves oxidative stress, anti-inflammatory compounds should be treated as antioxidants at the whole-body level [3,4]. The antioxidant effect depends on the system: aspirin or anti-inflammatory cytokines attenuate oxidative stress in the body but do not protect against the oxidation of oil during storage.

Plants are the primary source of natural antioxidants—compounds that exhibit antioxidant activity in vitro—and are extensively studied in more complex biological and food systems. Although antioxidants such as phenolic acids, flavonoids, or betalains are present in any organ of any plant, some plant materials are especially rich in antioxidants, and new antioxidant compounds are still isolated from various plant species. Medicinal plants used in different parts of the world are especially fascinating in this regard [5,6].

The antioxidant function is a primary function of some compounds (like glutathione) or is an accidental effect of a compound synthesized primarily for other (e.g., defensive) purposes. Therefore, it is no wonder that the ingested “antioxidant” exerts effects in the body that may not be related to its antioxidant activity, or, paradoxically, the antioxidant effect may result from the defensive reaction of the body. It should be remembered that an exogenous antioxidant, taken in milligram amounts and subject to metabolic modifications and elimination, cannot significantly modify the antioxidant potential of the body. However, stimulation of the body’s own antioxidant defense may be an important outcome [7]. Moreover, exogenous antioxidants may affect the activities of enzymes and signaling pathways in other ways, unrelated to their antioxidant activity. Therefore, elucidation of the mechanisms of action of ingested antioxidants at the cellular level is essential.

There are several fields of potential applications for natural antioxidants in biomedicine that spark great interest among researchers, such as neurodegenerative diseases. These diseases are associated with oxidative stress [8,9], and the administration of antioxidants able to penetrate the blood–brain barrier may attenuate this stress and have beneficial effects [10,11]. Another important field, partly overlapping with neurodegenerative diseases, is the attenuation of the inflammatory response. Aside from ameliorating oxidative stress, the ultimate effect of inflammation, antioxidants may interfere with pro-inflammatory signaling pathways and enzymes in the progression of inflammation [12,13,14,15].

Consumption of antioxidant compounds may affect the composition of the intestinal microbiome, which, in turn, can affect the antioxidant status of the organism [16,17]. These topics in antioxidant research are also addressed in the papers collated in this Special Issue.

## 2. An Overview of Published Articles

Several articles in this Special Issue are devoted to the mechanisms of action of natural antioxidants at a cellular level and in animal experiments.

Hong et al. (contribution 2) demonstrate that oral administration of epigallocatechin gallate (EGCG) for 7 days before anesthesia reduced neuronal damage and improved postoperative cognitive function in aged rats following general anesthesia with isoflurane. This effect was associated with modulation of microglial phenotype differentiation (lower M1 and higher M2 activation). These results suggest that EGCG can prevent postoperative cognitive dysfunction, likely by ameliorating neuroinflammation.

The paper by Liang et al. (contribution 3) explores the therapeutic effect of *Atractylodes macrocephala* Koidz. polysaccharide on the depression induced by pirarubicin chemotherapy in breast cancer mice. Administration of the polysaccharide was found to significantly improve depression-like behaviors in mice subjected to chemotherapy and reduce neuronal damage and inflammation in the hippocampus. Moreover, *Atractylodes macrocephala* polysaccharide altered the composition of the gut microbiota and intestinal metabolites in chemotherapy-treated mice. Depletion of gut microbiota through antibiotics diminished the effectiveness of the polysaccharide. The results of the study suggest that *Atractylodes macrocephala* polysaccharide alleviates depression-like behavior in chemotherapy-treated mice by regulating the gut microbiota and microbial metabolism, and by suppressing ferroptosis in intestinal tissues.

The article by Hua et al. (contribution 4) examines the potential of another antioxidant, cinnamaldehyde, a natural compound derived from *Cinnamomum osmophloeum* Kaneh, to prevent or mitigate intervertebral disk degeneration. This condition is caused by the progressive loss of extracellular matrix components in the nucleus pulposus, and is, i.a., a side effect of obesity. Elevated circulating leptin levels in obese individuals contribute to this degeneration by upregulating the expression of matrix metalloproteinase-1 (MMP-1). The authors show that cinnamaldehyde suppresses leptin-induced MMP-1 expression in SV40 human intervertebral disk cartilage endplate-derived stem cells and that this effect is due to inhibition of phosphorylation of the leptin receptor and STAT3 and blocking RhoA and NF-κB activation, without affecting JAK2 and ERK1/2 phosphorylation. These results suggest that cinnamaldehyde may be considered as a therapeutic agent for the intervertebral disk degeneration associated with obesity.

Lupeol, a triterpenoid present in various fruits, vegetables, and medicinal plants, has gained attention for its broad-spectrum antioxidant and anti-inflammatory activities. The paper by Saha et al. (contribution 6) describes the immunomodulatory and antioxidant effects of lupeol in human monocyte-derived dendritic cells exposed to 7-ketocholesterol as an oxidant. Lupeol preserved the immature, tolerogenic phenotype of dendritic cells by promoting a dose-dependent increase in the anti-inflammatory cytokine IL-10. This terpenoid also inhibited the upregulation of maturation markers (CD83, CD86), induced by 7-ketocholesterol, and suppressed the release of pro-inflammatory cytokines, IL-1β and IL-12p70. Lupeol-treated dendritic cells directed T cell polarization toward an anti-inflammatory and regulatory profile while dampening the inflammatory responses triggered by 7-ketocholesterol. Lupeol significantly increased nuclear NRF2 levels and activated the KEAP1-NRF2 signaling pathway. These findings suggest that Lupeol may be a promising, immunomodulatory agent that can be administered orally, promoting tolerogenic dendritic cells and offering potential applications in autoimmune and other chronic inflammatory diseases.

*Cinnamomum kanehirai* Hayata, known as the Taiwan camphor tree, contains active compounds with potential biomedical applications. Essential oils extracted from the twigs and leaves of *C. kanehirai* are known to exhibit antibacterial properties. However, the effects of these oils on the inflammatory response remained unexplored. The study by Liu et al. (contribution 7) shows that the essential oil derived from the heartwood of *C. kanehirai* exerts an anti-inflammatory effect by inhibiting the NLRP3, NLRP1, NLRC4, AIM2, and non-canonical inflammasomes in macrophages. The mechanisms underlying the anti-inflammatory activity of EOC were shown to involve a reduction in the production of ROS and NF-κB activation. The key anti-inflammatory compound present in the essential oil of the camphor tree seems to be terpinen-4-ol. The results of the study indicate the potential of *C. kanehirai* essential oil for future development.

Sometimes the effects of antioxidant compounds can, paradoxically, intensify oxidative stress, but this effect may be beneficial and potentiate anticancer therapy. Although hesperidin, a citrus flavonoid, was reported to alleviate cisplatin-induced oxidative injury in renal tissue, it was also found to amplify this injury in some malignant cells. The paper by Çınar et al. (contribution 1) demonstrates that hesperidin amplified cisplatin toxicity in laryngeal carcinoma (Hep-2) cells, acting as a pro-oxidant, promoting apoptosis through the accumulation of ROS, stimulation of lipid peroxidation, Ca^2+^ dysregulation, glutathione depletion, and mitochondrial depolarization. One of the mechanisms of hesperidin action was the stimulation of expression of the transient receptor potential melastatin-2 (TRPM2), promoting Ca^2+^ influx into the cells. This study highlights a promising therapeutic strategy consisting of the use of redox-active flavonoids in combination with conventional chemotherapeutics to reprogram tumor redox homeostasis.

Garlic is known to be rich in antioxidants, but is also cytotoxic, especially to cancer cells. The main reason of its cytotoxicity is the high content of organosulfur compounds, which are antioxidants but also deplete cells of antioxidants. The paper by Furdak et al. (contribution 11) presents the effects of garlic extracts on human erythrocytes as simple model cells. Garlic extract decreased the level of acid-soluble thiols, especially glutathione, induced a prooxidative shift in the cellular glutathione redox potential, oxidized hemoglobin to methemoglobin, increased the osmotic fragility of erythrocytes, induced hemolysis, augmented the level of ROS, and induced lipid peroxidation. These results help to understand the action of garlic on more complex cells.

Two papers of the Special Issue are devoted to the synthesis of new antioxidant compounds and the characterization of their properties.

Adenosine, one of the basic nucleosides, exhibits antioxidant and anticancer activity. An adenosine analog, 9-[trans-2,trans-3-dihydroxy-4-(hydroxymethyl)cyclopent-4-enyl]adenine ((neoplanocin A), NPA), a member of the carbocyclic nucleoside family, is a natural analog of adenosine isolated from the culture filtrate of the soil-dwelling fungus *Ampullariella regularis.* The modification of adenosine’s sugar framework resulted in the unique biological activity of natural (-)-neoplanocin A, including its broad antiviral and anticancer properties. The paper by Pawlowska et al. (contribution 8) compares the naturally occurring (-)-neoplanocin A and its synthetic analog, (+)-neoplanocin A, demonstrating that the natural stereoisomer is more cytotoxic than its synthetic analog. The results of this study indicate that both enantiomers prefer different targets: the anticancer mechanism of action of natural neplanocin A ((-)-NPA) seems to be connected with adenosine kinase activity, while the activity of its opposite enantiomer may be related to the interaction with adenosine deaminase.

Resveratrol is a promising neuroprotective agent against neurodegenerative disorders such as Alzheimer’s disease due to its significant antioxidant properties. Inhibition of acetylcholinesterase is thought to be another means of protection against the progression of Alzheimer’s disease. The paper by Mlakić et al. (contribution 10) describes the synthesis of 14 new heterocyclic analogs based on the biologically active *trans*-resveratrol using heterocyclic triphenylphosphonium salts and various benzaldehydes. These compounds combine the functions of antioxidants and acetylcholinesterase inhibitors, so they potentially offer double protection to Alzheimer’s patients. Molecular docking of selected compounds into cholinesterases was performed to visualize the structure of ligand–active site complexes and identify the non-covalent interactions responsible for the stability of these complexes. The in silico ADMET analysis selected the most promising compounds.

Two papers are devoted to the antioxidant composition of natural foods.

There is extensive evidence of the protective effects of pomegranate (*Punica granatum*) extracts. The pomegranate fruit consists of an outer, non-edible part and an inner part with several partitions separated by a soft, white membrane, containing seeds surrounded by translucent pulp. The paper by Maiuolo et al. (contribution 5) compares the composition and antioxidant activity of extracts obtained from whole fruit and the internal membrane extracts. The internal membrane extract exhibited higher antioxidant activity in several assays and was more effective at attenuating the increase in the ROS level induced by exogenous hydrogen peroxide in SH-SY5Y cells. These results suggest using the internal membrane extract rather than the whole fruit extract in further experiments on the beneficial health effects of pomegranate.

*Ulva lactuca* (sea lettuce) is used as a food additive and has many applications across the food, pharmaceutical, and cosmetic industries. The paper by Mutavski et al. (contribution 9) brings a detailed analysis of the composition of this microalga. The list of volatile compounds of sea lettuce includes various aldehydes, benzyl alcohol, (Z,Z,Z)-hexadeca-7,10,13-trienal, and hexadecanoic acid, all of which are used in the cosmetic industry. The high ratio of ω-3 to ω-6 polyunsaturated fatty acids is beneficial for health. The presence of carotenoids, especially β-carotene and lutein, and numerous phenolics makes sea lettuce a rich source of antioxidants.

## 3. Conclusions

Although fruits and such edible plants as sea lettuce are known to be rich sources of antioxidants, their detailed characterization can provide valuable information, concerning, e.g., the content of antioxidants in various parts of the pomegranate fruit or the ratio of ω-3 to ω-6 polyunsaturated fatty acids in sea lettuce.

The effects of natural antioxidants on the central nervous system and their anti-inflammatory effects are of continuous interest. EGCG may be effective in the prevention of postoperative cognitive dysfunction, especially in elderly individuals, most probably by ameliorating neuroinflammation; *Atractylodes macrocephala* Koidz. polysaccharide may mitigate depression induced by pirarubicin chemotherapy; lupeol may be a promising, immunomodulatory agent for potential applications in autoimmune and other chronic inflammatory diseases that can be administered orally, while cinnamaldehyde may be considered as a therapeutic agent for the intervertebral disk degeneration associated with obesity.

New knowledge on the mechanisms of action of natural antioxidants has been gathered. The anti-inflammatory activity of the essential oil derived from the heartwood of *C. kanehirai* was found to be mediated by inhibition of NLRP3, NLRP1, NLRC4, AIM2, and non-canonical inflammasomes; lupeol was demonstrated to increase nuclear NRF2 levels and activate the KEAP1-NRF2 signaling pathway; *Atractylodes macrocephala* polysaccharide was demonstrated to regulate the gut microbiota and suppress ferroptosis in intestinal tissues; EGCG was found to modulate microglial phenotype differentiation and garlic extracts to deplete cellular thiols and increase the level of intracellular ROS.

The synthesis of new antioxidants with double functions may lead to the discovery of new anticancer compounds and compounds that can more effectively combat Alzheimer’s disease by combining antioxidant activity and acetylcholinesterase-inhibiting activity.
